# Knockdown of hypoxia-inducible factor-1α accelerates peritoneal dissemination via the upregulation of MMP-1 expression in gastric cancer cell lines

**DOI:** 10.3892/etm.2012.600

**Published:** 2012-06-06

**Authors:** MASATSUGU HIRAKI, YOSHIHIKO KITAJIMA, KEITA KAI, JUN NAKAMURA, KAZUYOSHI HASHIGUCHI, HIROKAZU NOSHIRO, KOHJI MIYAZAKI

**Affiliations:** 1 Departments of Surgery and; 2 Pathology and Biodefence, Saga University Faculty of Medicine;; 3 Department of Surgery, National Hospital Organization Higashisaga Hospital;; 4 Saga University Faculty of Medicine, Saga, Japan

**Keywords:** hypoxia-inducible factor-1 α, gastric cancer, peritoneal metastasis, matrix metalloproteinase, matrix metallopeptidase 1

## Abstract

This study was performed to clarify the role of hypoxia-inducible factor-1 α (HIF-1α) in the development of peritoneal dissemination in a xenograft mouse model of gastric cancer. HIF-1α knockdown (KD) and control (SC) gastric cancer cells, which were established using the MKN45 and MKN74 cell lines, were studied. The two paired cell lines were directly inoculated into the peritoneal cavity of nude mice. The number and the weight of disseminated nodules were compared between tumors generated from the KD and SC cells. In addition, the molecular mechanism was addressed through analysis of the expression levels of metastasis-related genes. The MKN45-KD cell line demonstrated significantly greater numbers of disseminated nodules and formed a larger tumor mass than the MKN45-SC cell line (p<0.05). MKN74-KD cells also tended to induce a greater number of nodules and to produce those with a heavier weight than the SC cells. An *in vitro* adhesion assay revealed differing results regarding the adhesion activity to extracellular matrix and monolayer mesothelium cells of the gastric cancer cells derived from the various parental cells. However, the expression of MMP-1 mRNA in the disseminated nodules was significantly increased in the KD cells compared to the SC cells derived from the two parental cell lines (p<0.01). An immunohisto-chemical study further demonstrated that there was stronger staining for MMP-1 in the MKN74-KD in comparison to MKN74-SC cells. Loss of HIF-1α may contribute to the development of aggressive peritoneal dissemination via the upregulation of MMP-1 in gastric cancer cells.

## Introduction

Peritoneal metastasis is a common type of metastasis, and occurs in 34% of gastric cancer patients with recurrence, even following curative resection of the primary tumor (1,2). Peritoneal recurrence develops from micrometastasis, which is already seeded in the peritoneum prior to surgery and it is thought that there are also intraperitoneal free cancer cells exfoliated from the serosal surface of the primary tumor. A better understanding of the molecular mechanism underlying the systemic spread of gastric cancer, including its peritoneal dissemination, is essential to determine optimal therapeutic strategies.

Hypoxia is a hallmark of solid tumor formation, and is an independent prognostic factor in a diverse range of malignant tumors (3). Hypoxia is associated with local invasion, meta-static spread, resistance to radiotherapy and chemotherapy, and a poor prognosis in a number of human carcinomas. Hypoxia-inducible factor-1 α (HIF-1α) is a key transcription factor involved in the cellular response to hypoxic conditions, and is involved in angiogenesis, glycolysis, the control of vascular tone and erythropoiesis (3). HIF-1 is a heterodimeric protein consisting of a constitutively expressed β subunit and the HIF-1α subunit. Previous reports have demonstrated that upregulation of HIF-1α occurs in cancer cells subjected to hypoxia, that there is a hypoxia/HIF-1α cascade that is activated in solid cancer, and that there are relationships between the upregulation of HIF-1α and a poor prognosis and chemo-resistance in gastric cancer (3,4). However, little is known concerning the mechanism and contributory roles of HIF-1α expression and the development of peritoneal dissemination in gastric cancer.

We previously established two gastric cancer cell lines with HIF-1α knockdown (KD), MKN45-KD and MKN74-KD (4), and demonstrated the involvement of HIF-1α expression in chemoresistance using nude mouse xenograft models. In the present study, we first compared the development of peritoneal dissemination in the peritoneal cavity of nude mice between HIF-1α KD and control (SC) gastric cancer cell lines. Then, we elucidated the molecular mechanism underlying this process by investigating changes in the expression of metastasis-related genes between the KD and SC cells. This study was performed to clarify whether or not HIF-1α expression affects the development of peritoneal dissemination and to isolate candidate genes regulated by HIF-1α that may be involved in peritoneal dissemination.

## Materials and methods

### Cell lines and culture

Two gastric cancer cell lines, MKN45 and MKN74, were purchased from Riken Cell Bank (Ibaraki, Japan). MKN45 was derived from a poorly differentiated adenocarcinoma and MKN74 was derived from a moderately differentiated adenocarcinoma. To analyze the role of HIF-1α in peritoneal dissemination, we established two HIF-1α knockdown sublines (designated MKN45-KD and MKN74-KD, respectively) and control sublines (MKN45-SC and MKN74-SC, respectively) as reported in a previous study (4). These four gastric cancer cell lines, MKN45-SC, MKN45-KD, MKN74-SC and MKN74-KD, were used for this study. The cell lines were cultured as described in a previous study (4).

### Animal experiments

The animal protocols were approved by the Animal Care Committee of Saga University. Female athymic BALB/cA Jcl mice (nu/nu, 5-weeks old) were obtained from Nihon Crea Co. (Osaka, Japan). They were maintained under specific pathogen-free conditions and were given γ-irradiated food and autoclaved water. We examined the peritoneal dissemination using the four gastric cancer cell lines, MKN45-SC and -KD, and MKN74-SC and -KD. To generate the xenograft model, cancer cells (5x10^6^) were suspended in 100 μl of PBS, and injected on day 0 into the abdominal cavity. Five mice per group were injected with the various cell lines. All of the mice were sacrificed on day 28, and then the number of disseminated nodules and their total weight were measured.

### Western blot analysis

The western blot analysis was performed as previously described (4). In brief, the whole cell lysate was prepared using a lysis buffer composed of 150 mM NaCl, 50 mM Tris-HCl (pH 7.6), 0.5% Triton X-100 and a protease inhibitor cocktail mix (Roche, Mannheim, Germany). The samples were dissolved in NuPage™ LDS sample buffer (Invitrogen, Carlsbad, CA, USA) and 1 M dithiothreitol. A total of 20 μg of protein was subjected to NuPage 4–12% Bis-Tris Gel (Invitrogen) electrophoresis and electrophoretically transferred onto an Amersham™ Hybond™-ECL membrane (GE Healthcare, Buckinghamshire, UK) in transfer buffer. The primary antibodies used in this analysis were anti-HIF-1α (BD Biosciences, San Jose, CA, USA) and anti-β-actin (Sigma). Following incubation with the corresponding secondary antibodies, the signals were developed using an Amersham™ ECL Plus Western Blotting Detection System (GE Healthcare).

### Total RNA extraction and real-time quantitative reverse transcription-polymerase chain reaction

Total RNA was extracted using an Isogen^®^ RNA extraction kit (Nippon Gene, Osaka, Japan), converted to cDNA using a ReverTra Ace reverse transcription reaction kit (Toyobo, Osaka, Japan) and subjected to real-time quantitative reverse transcription (RT)-PCR using Light Cycler FastStart DNA Master™ SYBR Green I kit and a Light Cycler™ instrument system (Roche) (4). The mRNA expression levels of HIF-1α, CA9 and metastasis-related genes, such as integrin α2, α3, α5, α6, αv, β1, β3, β4, β5, β6, matrix metallopeptidase (MMP)-1, MMP-7, MMP-11, α-catenin, β-catenin, CD44 and E-cadherin, were examined. The primer sequences used are shown in Table I.

### Adhesion assay

To quantify the tumor cell adhesion to the extracellular matrix and the monolayer mesothelium, an adhesion assay was performed. The *in vitro* adhesion assay was carried out using the CytoSelect 48-well cell adhesion assay extracellular matrix (ECM) array (Cell Biolabs, Inc, San Diego, USA) according to the manufacturer’s instructions. Another adhesion assay using the monolayer mesothelium was performed as described below. The mesothelial cell line, MeT-5A (CRL-9444), was purchased from the American Type Culture Collection (Manassas, VA, USA). A total of 2x10^4^ MeT-5A cells were added in 100 μl of medium to each well of a 96-well plate. The plate was incubated at 37°C under normoxic conditions overnight. Then a mesothelial monolayer was established. Each well was washed with PBS to remove non-adherent cells, then 2x10^4^ cells of each gastric cancer cell line were added to 100 μl of medium to each well. The plate was then incubated at 37°C under normoxic conditions for 5 h. Each well was washed with PBS to remove non-adherent cells, and then the MTT assay was performed using a Cell-Titer 96™ nonradioactive cell proliferation assay kit (Promega, Madison, WI, USA). The adhesion index of the mesothelium was calculated using the following formula: Adhesion index = (Adherent cells in well)/(Seeded cells in well).

### Immunohistochemistry

The immunohistochemical staining of MMP-1 and MMP-11 was performed according to a previous report (4). The anti-MMP-1 antibody (Thermo Scientific, Fremont, CA, USA) and anti-MMP-11 antibody (Abcam, Tokyo) were used as the primary antibodies.

### Statistical analysis

The statistical analysis was carried out using the SPSS 1.5 statistical software package for Windows (SPSS Japan Inc.) A P-value <0.05 was considered to be indicative of statistical significance.

## Results

### Characteristics of the development of peritoneal dissemination dependent on HIF-1α in xenograft tumors in nude mice

**Figure 1:**
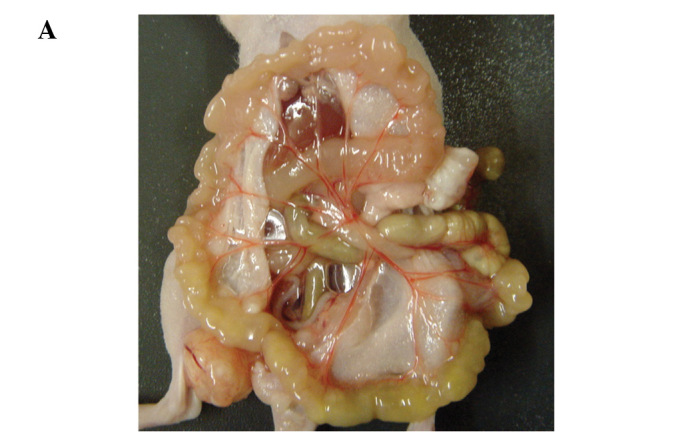

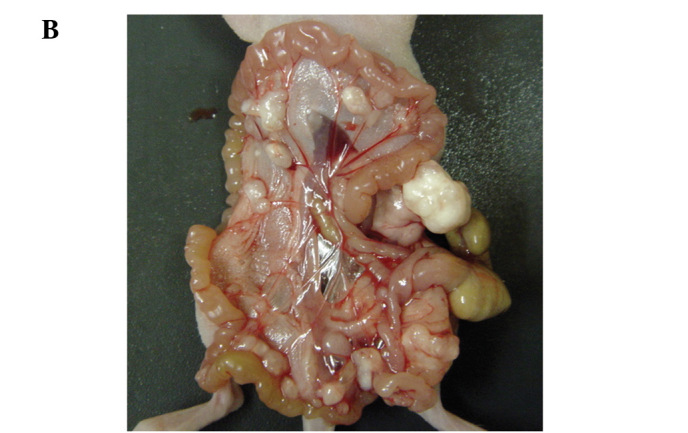

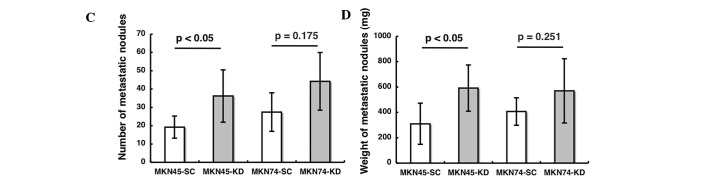

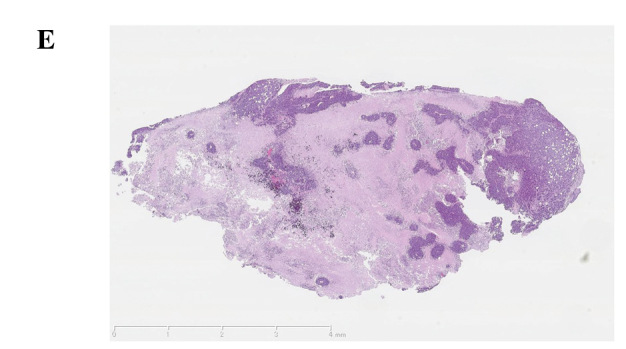

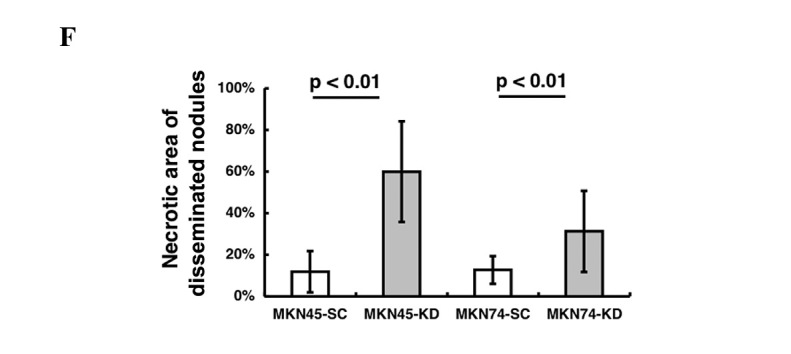
(A) Metastatic nodules derived from MKN45-SC and (B) MKN45-KD cells in nude mice. (C) Total number of metastatic nodules. (D) Total weight of metastatic nodules. (E) Necrosis in a dissemination nodule of nude mouse tissue derived from MKN45-KD cells. (F) Necrotic area of disseminated nodules. The MKN45-KD cells developed more disseminated nodules than did the MKN45-SC cells. Both types of KD cells (MKN45 and MKN74) showed a higher number of disseminated nodules and a heavier weight than the SC cells. The microscopic examination showed that the disseminated nodules derived from the KD cells had a larger necrotic area than that of the SC cells.

All four gastric cancer cell lines, MKN45-SC, -KD, MKN74-SC, -KD, resulted in peritoneal dissemination in nude mice (5 of 5; 100%, respectively). The differences in the development of peritoneal dissemination between the SC and KD cell lines were compared using the MKN45 and MKN74 cells. Regarding the MKN45 cell lines, the HIF-1α KD cell line resulted in significantly greater numbers of disseminated nodules and a heavier weight of nodules than the SC cell lines (p<0.05, p<0.05, respectively, Fig. 1A–D). In the MKN74 cell lines, HIF-1α KD also tended to result in greater numbers of disseminated nodules and a heavier weight of nodules than the SC cells, although there were no significant differences (p=0.175, p=0.251, respectively, for the number and weight; Fig. 1C and D). Microscopic observation revealed that the disseminated nodules in the KD cells tended to have a larger necrotic area than the SC cells (Fig. 1E). The area of necrosis in the largest disseminated nodule in each mouse was estimated, and the area of dissemination of the two KD cell lines was found to be significantly larger than that of the SC lines (Fig. 1E and F, p<0.01).

### HIF-1α siRNA significantly decreases the expression of HIF-1α and its target gene in xenograft tissues

**Figure 2:**
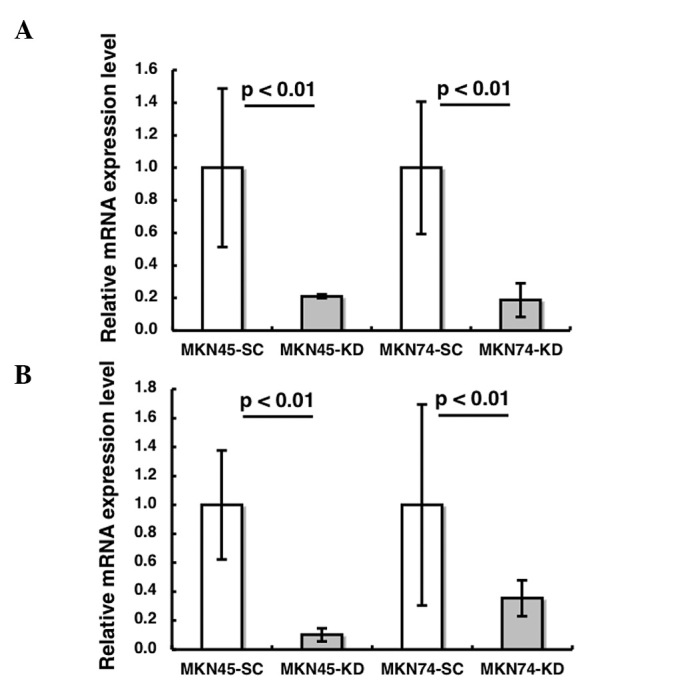

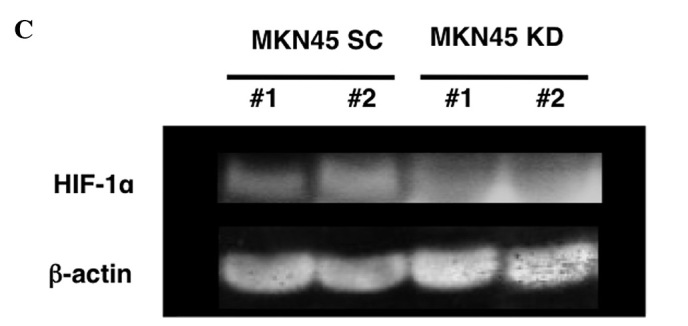
(A) HIF-1α mRNA expression in nude mouse tissues. (B) CAIX mRNA expression in nude mouse tissues. (C) HIF-1α protein expression in nude mouse tissues. The mRNA expression levels of HIF-1α and CAIX were significantly reduced in KD tissues compared to SC tissues. The protein expression of HIF-1α was undetectable in the disseminated nodules derived from MKN45-KD cells.

The induction of HIF-1α and its target gene, CAIX, were validated by RT-PCR and/or immunoblotting in nude mouse tissues. The mRNA expression levels of HIF-1α and CAIX were significantly reduced in the HIF-1α KD tissues compared to the SC tissues (p<0.01, p<0.01, respectively Fig. 2A and B). The immunoblotting analysis demonstrated that HIF-1α was undetectable in HIF-1 KD tumors, whereas HIF-1α expression was observed in SC tumors (Fig. 2C).

### Adhesion to the extracellular matrix or a monolayer mesothelium

**Figure 3:**
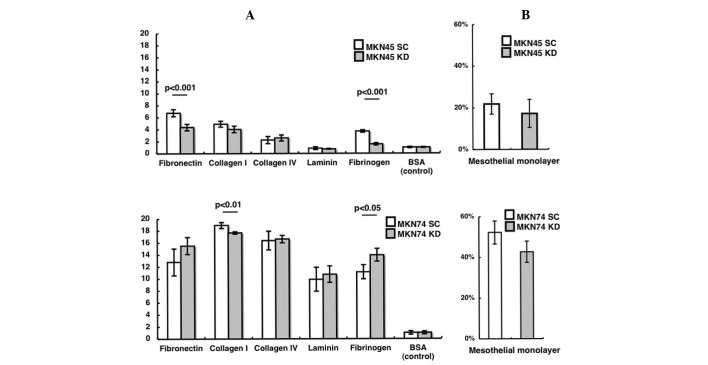
Results of adhesion assays. (A) In the MKN45 cells, attachment to fibronectin and fibrinogen was significantly decreased in the KD compared to the SC cell line. In the MKN74 cells, attachment to collagen I was significantly decreased and that to fibrinogen was significantly increased in the KD compared to the SC cell line. (B) In terms of adhesion to the mesothelial monolayer, no significant difference was found between the KD and SC sublines for both the MKN45 and MNK74 cell lines.

To investigate why the HIF-1α KD cell lines develop a greater number of peritoneal nodules than the SC cell lines, an adhesion assay was performed. In the MKN45 cells, attachment to fibronectin and fibrinogen was significantly decreased in the KD compared to the SC cell line (Fig. 3A; p<0.001, P<0.001, respectively). In the MKN74 cells, attachment to collagen I was significantly decreased and that to fibrinogen was significantly increased in the KD compared to the SC cell line (Fig. 3A; P<0.01, P<0.05, respectively). In terms of adhesion to the mesothelial monolayer, no significant difference was found between the KD and SC sublines for both the MKN45 and MNK74 cell lines (Fig. 3B).

### mRNA expression of metastasis-related genes in the various cell lines

To elucidate why HIF-1α KD cells demonstrated an increased number of disseminated nodules compared to the SC cells, the mRNA expression levels of metastasis-related genes were analyzed in each cell line. We investigated the mRNA levels in the two paired SC and KD cell lines under normoxic and hypoxic conditions and subsequently estimated the expression ratios of the KD/SC cells. This analysis isolated a candidate gene, which was commonly upregulated in the KD cells of the MKN74 and MKN45 cell lines, compared with the SC cells. In the MKN45 cells, the expression level of MMP-1 was higher in the KD than SC cells under both normoxic (KD/SC ratio: 2.88) and hypoxic (KD/SC ratio: 1.96) conditions (Table II). In the MKN74 cells, the higher ratio was also observed under both normoxic (KD/SC ratio: 3.86) and hypoxic conditions (KD/SC ratio: 3.23). MMP-11 was more highly expressed in KD than in SC MKN45 cells (KD/SC ratio: 1.58 under normoxia, 1.70 under hypoxia) and MKN74 cells (KD/SC ratio: 10.70 under normoxia, 4.32 under hypoxia). On the other hand, CD44, catenin β1, integrin α5 and integrin β4 demonstrated higher mRNA expression under both conditions of normoxia and hypoxia in the MKN74 cells. However, higher expression of these genes was not observed in the MKN45 cells. Taken together, the results suggest that MMP-1 and MMP-11 are commonly upregulated in both KD cell lines under normoxic and hypoxic conditions.

### mRNA and protein expression of MMP-1 and -11 in disseminated nodules in nude mice

**Figure 4:**
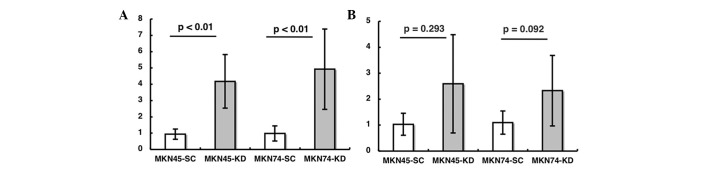

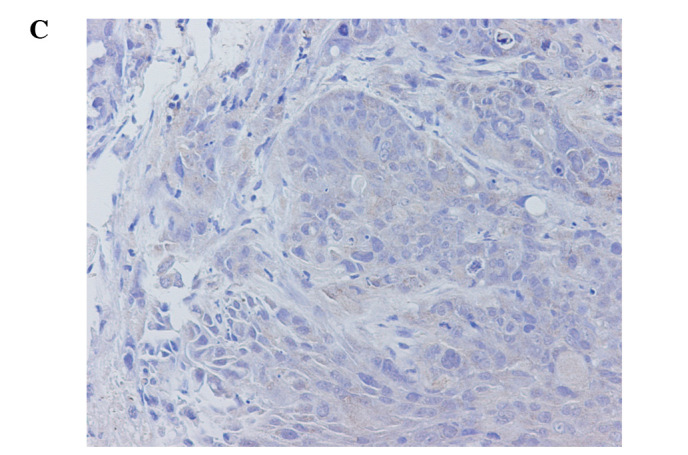

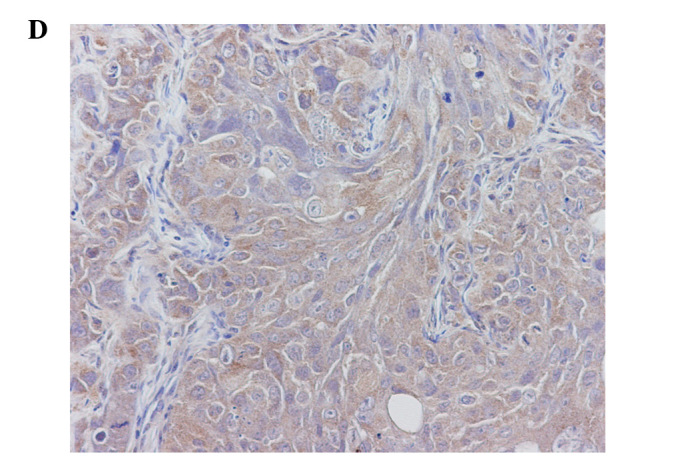
(A) MMP-1 mRNA expression in nude mice tissues. (B) MMP-11 mRNA expression in nude mice tissues. (C) MMP-1 expression in MKN74-SC tissue. (D) MMP-1 expression in MKN74-KD tissue. The mRNA expression of MMP-1 in the disseminated nodules was significantly increased in the KD sublines than the SC sublines of both parental cell lines. The mRNA expression of MMP-11 in the disseminated nodules showed a tendency to be increased in the KD cells compared to the SC cells. An immunohistochemical study showed that there was significantly stronger staining for MMP-1 in the MKN74-KD to that in the MKN74-SC cells.

The mRNA expression level of MMP-1 in the disseminated nodules in nude mice was analyzed. The expression was significantly increased in the nodules derived from the KD sublines compared to those from the SC sublines for the MKN45 and MKN74 cell lines (Fig. 4A; p<0.01, p<0.01, respectively). The mRNA expression of MMP-11 in nude mouse tissues tended to be increased in the KD compared to the SC sublines, although the difference was not significant (Fig. 4B; p=0.293, p=0.092). An immunohistochemical study of MMP-1 in nude mouse tissues demonstrated that there was significantly stronger staining in MKN74-KD than in MKN74-SC cells (Fig. 4C and D). However, the MMP-11 staining in the MKN74 cells did not demonstrate any significant difference between the SC and KD cells (data not shown).

## Discussion

Peritoneal metastasis is the most common cause of tumor progression in gastric cancer. When the dissemination of multiple peritoneal nodules are obvious, curative surgery with a standard gastrectomy cannot be achieved. In addition, peritoneal metastasis exhibits resistance against various chemotherapeutic drugs, and causes massive ascites and intestinal obstruction. A previous study reported that 50–60% of gastric cancer patients with serosal invasion following curative resection eventually developed peritoneal metastasis (5), and the average survival following peritoneal recurrence was only 4.9 months (6). Therefore, clarifying the mechanism underlying the peritoneal metastasis is essential not only for preventing the formation of gross metastatic nodules in the peritoneum, but also for considering the treatment strategy.

As a key regulator of the cellular adaptive response to hypoxia, HIF-1α plays a critical role in tumorigenesis (3). Hypoxic cancer cells may undergo a series of genetic and metabolic changes that allow them not only to survive and proliferate, but also to become more resistant to radiation therapy and chemical agents. The formation of metastases is a major factor in disease progression. In the present study, we silenced the function of HIF-1α to evaluate its role in the peritoneal dissemination of gastric cancer cell lines. The molecular mechanism underlying the peritoneal dissemination of cancer cells remains poorly understood and, so far, there have been no prognostic markers indicating which primary gastric tumors are likely to develop peritoneal dissemination.

As previously reported, the protein expression of HIF-1α in MKN45-KD and MKN74-KD cells was undetectable even following hypoxic stimulation, and the mRNA expression of its target genes, GLUT1 and CA9, was significantly suppressed in comparison to the SC cells (4). We also confirmed that the HIF-1α and CA9 mRNA expression levels were significantly reduced in the disseminated nodules derived from the two KD cell lines (Fig. 2A and B), and the HIF-1α protein was not detectable in the disseminated nodules of either of the KD cell lines (Fig. 2C).

We made the unexpected observation that the loss of HIF-1α accelerates the peritoneal dissemination of MKN45 and MKN74 cell lines. This was contrary to our expectation, since several previous studies had demonstrated that HIF-1α is involved in cell proliferation and/or tumorigenesis in gastric cancer cell lines (7), colorectal cancer cell lines (8), endothelial cell lines (9) and chondrocytes (10). However, a few studies have described the tumor-suppressor functions of HIF-1α. For example, Carmeliet *et al* (11) reported that the loss of HIF-1α led to more aggressive growth of embryonic stem cell-derived teratocarcinomas. Blouw *et al* (12) reported that astrocytomas deficient in HIF-1α grow faster, and penetrate the brain more rapidly and extensively than their normal counterparts. We also previously reported that HIF-1α KD MKN45 tumors in xenografts grown in nude mice demonstrated more rapid growth, in comparison to the control MKN45 tumors (4). Our results and previous reports suggest that HIF-1α expression may act as a negative factor in the formation of metastasis under certain conditions, such as subcutaneous and peritoneal cavity growth.

The mechanism of peritoneal dissemination of gastric cancer has been described to occur through several steps; detachment of cancer cells from the primary tumor, attachment to the distant peritoneum, invasion into the subperitoneal space and proliferation with vascular neogenesis (13). Regarding attachment to the peritoneum, the adhesion assay for the extracellular matrix and mesothelial cells was performed. We found that the attachment to fibronectin and fibrinogen in the MKN45 cells and collagen I in the MKN74 cells were significantly decreased in KD cells in comparison to SC cells. On the other hand, in the MKN74 cells, only fibrinogen was significantly increased in the KD cells compared to the SC cells. These results indicated that cell attachment molecules have little impact on the development of dissemination related to HIF-1α expression.

In order to further clarify why the KD cell lines demonstrated more peritoneal dissemination than the SC cell lines, we next compared the mRNA expression level of metastasis-related genes, such as integrin family member MMP, α and β catenin, CD44 and E-cadherin, between the KD and SC cell lines. In this analysis, the mRNA expression levels of MMP-1 and MMP-11 were significantly upregulated in the HIF-1α KD cell lines under both normoxic and hypoxic conditions, compared with the SC cells (Table II). In addition, the protein expression of MMP-1, but not MMP-11, was dramatically increased in the MKN74-KD cells in comparison to the MKN74-SC cells (Fig. 4C). These results indicated that the effects on MMP-1 may be the most critical effect mediated by the loss of HIF-1α expression.

Matrix metalloproteinases (MMPs) are believed to mediate a number of physiological and pathological processes, such as the degradation of the extracellular matrix, tissue remodeling, inflammation, tumor invasion and metastasis. The present study focused on the expression of MMP 1, 7 and 11, since several studies previously reported the relationships between the MMP members and invasion or dissemination in gastric cancer (14–21). For example, Yanagihara *et al* (22) reported that a cDNA microarray analysis demonstrated dramatic upregulation of MMP-1 (ratio: 29.63) in the 44As3 gastric cancer cell subline which shows frequent metastasis to the peritoneum, in comparison to parental HSC-44PE cells. In addition, previous studies reported that an MMP inhibitor reduced the peritoneal dissemination of human gastric cancer cell lines in mice (18,23). Taken together, our results and the previous findings indicate that the upregulation of MMP-1 expression following the loss of HIF-1α might be an important step in the development of peritoneal dissemination from MKN45 and MKN74 gastric cancer cells.

Knocking down HIF-1α, which leads to MMP-1 upregulation, might contribute to the degradation of the extracellular matrix of the peritoneum, allowing the invasion of cancer cells and the formation of peritoneal metastasis. However, contrary to this result, Miyoshi *et al* reported that hypoxic stress accelerated cancer invasion by upregulating the expression levels of MMP-7 and -14 by a HIF-1α-independent pathway (24). Fujiwara *et al* demonstrated that treatment with HIF-1α siRNA resulted in the downregulation of MMP-2 mRNA under hypoxic conditions in all of the glioma cell lines examined (25). Considering these controversial findings, it is possible that each member of the MMP family might play a different role in cancer progression, such as cancer invasion by the primary tumor, distant metastasis and peritoneal dissemination.

In conclusion, the present study demonstrates for the first time that the loss of HIF-1α contributes to the development of aggressive peritoneal dissemination by upregulating MMP-1 in gastric cancer cell lines. Therefore, HIF-1α expression might be suppressed during the development of peritoneal dissemination from primary gastric cancer.

## Figures and Tables

**Table I:** Primer sequences used in quantitative real-time PCR.

Gene symbol	Forward primer	Reverse primer
CA9	CCGAGCGACGCAGCCTTTGA	GGCTCCAGTCTCGGCTACCT
CD44	CAGCACCATTTCAACCACAC	AGCACTTCCGGATTTGAATG
CDH1	CTGAAAGCGGCTGATACTGAC	GGAGTTCAGGGAGCTCAGACT
CTNNA1	CCCAAGTTTTCCGTGAACAT	GCTTGCAGACATTCGAACAA
CTNNB1	ACCTTTCCCATCATCGTGAG	AATCCACTGGTGAACCAAGC
HIF-1α	CTCATCAGTTGCCACTTCCA	CCTCACACGCAAATAGCTGA
ITGA2	TGTCCTGTTGACCTATCCACTG	AGGCTCATGTTGGTTTTCATCT
ITGA3	CTACCACAACGAGATGTGCAATA	ATCATGTAGCTGTTTCCTTTCCA
ITGA5	TGTTGGTGAATTCAGTGGTGA	GAGCCATTAAGGATGGTGACA
ITGA6	GCGAGCAAGCTATGAAATCTG	CTGTGCCGAGGTTTGTAAGAG
ITGAV	ATCTGTGAGGTCGAAACAGGAT	ATCCGAAATAAGCTGACGTGAT
ITGB1	TACTTGTGAAGCCAGCAACG	CACGTTTGCCCTTGAAACTT
ITGB3	TCAATGAGGAAGTGAAGAAGCA	GTCTTGGCATCAGTGGTAAACA
ITGB4	ACCCAGTACAGGACACAGGACTA	AGGAGTAGTTGGTGACAGCAAAG
ITGB5	TGCAGCACCAAGAGAGATTG	CTCATCCCTGCATAGGCTGT
ITGB6	AATGACTCCCTCCACCTCCT	TGCTGTCCAAGTGACAGAGC
MMP1	AGGTCTCTGAGGGTCAAGCA	TCCTCCAGGTCCATCAAAAG
MMP7	AGCTCATGGGGACTCCTACC	GTGAGCATCTCCTCCGAGAC
MMP11	CCGCCTCTACTGGAAGTTTG	GCACAGCCAAAGAAGTCAGG
ACTB	CGAGCGCGGCTACAGCTT	TCCTTAATGTCACGCACGATTT

**Table II:** Relative differences in gene expression in the cell lines under conditions of normoxia and hypoxia.

		MKN45-KD/MKN45-SC		MKN74-KD/MKN74-SC	
Gene symbol	Gene description	Nx	Hx	Nx	Hx
CD44	CD44 molecule	1.34	0.72	**25.74**	**5.59**
CDH1	E-cadherin	0.98	0.75	0.52	1.00
CTNNA1	Catenin α1	1.06	0.78	1.00	1.08
CTNNB1	Catenin β1	0.70	0.75	1.67	**2.40**
ITGA2	Integrin α2	1.38	0.48	0.51	0.52
ITGA3	Integrin α3	0.56	0.40	1.07	0.68
ITGA5	Integrin α5	0.84	0.55	1.75	1.64
ITGA6	Integrin α6	1.65	0.83	1.40	**3.09**
ITGAV	Integrin αV	1.04	1.21	0.75	**2.22**
ITGB1	Integrin β1	1.35	1.49	1.09	1.58
ITGB3	Integrin β3	0.79	1.01	**7.62**	0.89
ITGB4	Integrin β4	1.54	0.65	1.59	1.84
ITGB5	Integrin β5	1.67	1.29	0.91	1.06
ITGB6	Integrin β6	0.81	0.68	0.93	1.49
MMP1	Matrix metallopeptidase 1	**2.88**	1.96	**3.86**	**3.23**
MMP7	Matrix metallopeptidase 7	0.09	0.13	1.56	1.01
MMP11	Matrix metallopeptidase 11	1.58	1.70	**10.70**	**4.32**

Nx, normoxia; Hx, hypoxia. Underlined print indicates a >1.5-fold induction and bold print indicates a >2.0-fold induction.
